# Anti-Typhus Appeal

**Published:** 1921-01-15

**Authors:** 


### Anti-Typhus Appeal.

A TELEGRAPHIC appeal to all members of the League of Nations to make every possible effort to contribute generously and immediately towards the fund of £2,000,000 required to combat the typhus epidemic in Eastern Europe, beginning with Poland as a centre, has just been sent out in conformity to the resolution of the First Assembly. It is hoped that this appeal may bring early action from those nations which have not yet contributed to the fund, in order to enable the campaign to be started before the typhus situation has become any more desperate. Typhus is practically unknown now in this country to the general public. Yet there was a time when this dread disease, the companion of neglect, dirt, and semi-starvation, was a too common destroying agent. Mr. Thomas Johnston, who has just published an interesting and valuable "history of the working classes in Scotland," points out somewhere in that book that in the first forty years of the nineteenth century 350,000 dwellers on the banks of the Clyde suffered periodic decimation from typhus, one writer estimating a constant residue of 30,000 widows and orphans. The danger of a serious outbreak in Great Britain is no doubt remote in these days; but it is nevertheless in the material interest of the people of these islands, apart altogether from considerations of humanity, to contribute to this fund for Eastern Europe. Typhus has an extraordinary devitalising effect on its victims, and when it is widespread its effect may be marked on the productive capacity of a whole nation, or over a very large area of its populous districts, a fact already noted in Poland and other parts of Eastern Europe. It is obvious, therefore, even if the danger does not extend westward to any large extent, that the economic condition of the countries involved must be very seriously affected, and that in turn the reaction must be increasingly felt in an industrial and exporting country like ours. There are many very good reasons why Great Britain should respond generously and promptly to the League's appeal.

